# Supplementary material for: Improved physical performance in obesity-resistant rats compared to obesity-prone rats: Effects of different diets and metabolic analysis
Source: PLoS One. 2025 Jul 7;20(7):e0327670. doi: 10.1371/journal.pone.0327670 (PMC12233250; doi:10.1371/journal.pone.0327670)
Supplement: S1 Table — *Standard diet for rodents. BHT – Butylated Hydroxytoluene. (PDF) [file pone.0327670.s001.pdf]

Supporting Information Citations

S1. Composition of macronutrients in the standard diet and high-fat diet.

Components		
	Dieta Nuvilab CR-1* (g)	High-Fat diet (g)
**Carbohydrates (g)**	60	49
**Protein (g)**	22	17
**Lipids (g)**	6,67	24
**Crude Fiber (g)**	5,0	5,0
**Vitamin Mix**	2,5	1,0
**Mineral Mix**	2,13	3,5
**Amino Acids**	1,60	0,16
**BHT**	0,10	0,25
**Total (g)**	**100**	**100**

.*Standard diet for rodents. BHT - Butylated Hydroxytoluene.
