# Supplementary material for: Improved physical performance in obesity-resistant rats compared to obesity-prone rats: Effects of different diets and metabolic analysis
Source: PLoS One. 2025 Jul 7;20(7):e0327670. doi: 10.1371/journal.pone.0327670 (PMC12233250; doi:10.1371/journal.pone.0327670)
Supplement: S2 Table — *Standard diet for rodents. Parte superior do formulário. (PDF) [file pone.0327670.s002.pdf]

Supporting Information Citations

S2. Macronutrient percentages and caloric density.

**Components**	Diets	
	**Nuvilab CR-1***	**High-Fat diet**
Carbohydrates (%)	61,86	40,29
Protein (%)	22,68	14,38
Lipids (%)	15,46	45,33
**Caloric density (kcal/g)**	**3,88**	**4,83**

*Standard diet for rodents. levin
