# Supplementary material for: Improved physical performance in obesity-resistant rats compared to obesity-prone rats: Effects of different diets and metabolic analysis
Source: PLoS One. 2025 Jul 7;20(7):e0327670. doi: 10.1371/journal.pone.0327670 (PMC12233250; doi:10.1371/journal.pone.0327670)
Supplement: Attachment — Submitted filename: Response to Reviewers.docx [file pone.0327670.s004.docx]

**Plos One**

| **Subject:** | PONE-D-24-57591 |
| --- | --- |
| **By**: | Daniel Sesana da Silva, Matheus Corteletti dos Santos, Lucas Furtado Domingos, Jóctan Pimentel Cordeiro, Kiany Miranda, Maria Gabriela Siqueira Tavares, Késsia Cristina Carvalho dos Santos, Ana Paula Lima-Leopoldo, André Soares Leopoldo |
| **Title**: | Improved Physical Performance in Obesity-Resistant Rats Compared to Obesity-Prone Rats: Effects of Different Diets and Metabolic Analysis |

**Comments to the Author**

**Reviewer #1: The study has a major problem. The aim was comparison of obesity-prone (OP) vs. obesity-resistant (OR) rats, and any interpretation and discussion builds on the assumption that this was done. However, the employed protocol did not deliver groups of OP and OR rats. Furthermore, the HFD exerted very different action vs. HFD used in other studies, which hampers interpretation in the light of previous studies.**

**It appears that this study did NOT compare obesity-prone vs. obesity-resistant rats as the authors claim. Obesity is a state of excessive fat accumulation, but the “adiposity index” indicative of relative fat mass is not different between the OP and OR rats. Hence, lean and fat mass are increased proportionally in the “OP” vs. the “OR” groups examined in this study, which means that the authors have actually compared rats that grew bigger vs. smaller, without any difference in their proneness to develop obesity.**

Dear Reviewer, we sincerely appreciate your insightful comment, which we consider extremely pertinent to the discussion regarding the criteria used for the identification of obesity resistance. Indeed, we acknowledge that obesity, from a pathophysiological standpoint, should be characterized by the excessive accumulation of adipose tissue. However, body mass has been commonly employed to identify OP (obesity-prone) and OR (obesity-resistant) phenotypes, particularly in protocols aimed at investigating susceptibility to diet-induced obesity. Although this criterion presents limitations in terms of accurately characterizing obesity per se, it has been widely used in several studies with the specific objective of distinguishing animals with greater or lesser propensity for body mass gain under identical dietary exposure.

The use of body mass has proven effective in differentiating OP and OR groups in experimental studies since the seminal work of Levin, Triscari, and Sullivan (1983). In this context, stratification based on tertiles of body mass distribution has been a commonly adopted approach in the literature: animals in the upper tertile are classified as OP, while those in the lower tertile are considered OR (1-3)

In the current study, group stratification was performed prior to euthanasia, at the third week of dietary intervention, at which point body composition or fat depot data were not yet available. Thus, this can lead to problems in the composition of the groups and interfere with adiposity gain. In addition, we recognize that the findings obtained were unexpected, particularly considering previous results from our research group, where we observed significant differences in adipose depot composition and adiposity index between OP and OR groups (4-5). In this sense, we believe that the comparison of body mass, as well as previous studies highlighted, can be considered. It should be noted that we also carried out histological analyses of visceral adipose tissue (VAT) and observed changes (OR vs. OP in High-fat diet; new figure 4) that could support this comparison between obesity-prone vs. obesity-resistant rats. Therefore, we opted to perform morphological analyses given that visceral fat accumulation is a known risk factor for mortality. This information was added in Material and Methods section ***(Histological Analysis of Visceral Adipose Tissue***, ***page 10),*** as well as ***Results (page 14).***

1. BROWN, R. M.; KUPCHIK, Y. M.; SPENCER, S.; GARCIA-KELLER, C.; SPANSWICK, D. C.; LAWRENCE, A. J.; SIMONDS, S. E.; SCHWARTZ, D. J.; JORDAN, K. A.; JHOU, T. C.; KALIVAS, P. W. Addiction-like Synaptic Impairments in Diet-Induced Obesity. **Biol Psychiatry**, v. 81, n. 9, p. 797-806, 2017/00 2017.
2. CLUNY, N. L.; ELLER, L.K.; KEENAN, C. M.; REIMER, R. A.; SHARKEY, K. A. Interactive effects of oligofructose and obesity predisposition on gut hormones and microbiota in diet-induced obese rats. **Obesity (Silver Spring)**, v. 23, n. 4, p. 769-778, 2015/03 2015.
3. LU, Y.; LI, Y.; SUN, Y.; MA, S.; ZHANG, K.; TANG, X.; CHEN, A. Differences in energy metabolism and mitochondrial redox status account for the differences in propensity for developing obesity in rats fed on high-fat diet. **Food Sci Nutr**, v. 9, n. 3, p. 1603-1613, Mar 2021
4. PIMENTEL CORDEIRO, J. et al. Resistance to obesity prevents obesity development without increasing spontaneous physical activity and not directly related to greater metabolic and oxidative capacity. **Plos one**, v. 17, n. 8, p. e0271592, 2022.
5. CARDOSO, J. C. et al. A high-fat diet induces cardiac damage in obesity-resistant rodents with reduction in metabolic health. **Cell. Physiol. Biochem**, v. 57, p. 264-278, 2023.

**The effects of the HFD formulation used in this study had very different effects on Wistar rats vs. HFD used in previous studies. Albeit HFD caused a (marginal) increase in body weight vs. SD after the initial 3 weeks, this was abolished after 23 weeks, which clearly differs from the effects of HFD used in other studies, which has been described to cause a marked and persistent increase in the body weight of Wistar rats. The reason for this difference is unclear (low palatability of HFD used here?). Interpretation and discussion in the paper do not address this matter, but are rather based on the assumption that HFD used here is the same as HFD used by others.**

Dear Reviewer, thank you for your insightful comment. We acknowledge that animals subjected to the high-fat diet (HFD) exhibited lower food consumption and caloric intake, and we recognize that this perspective was not clearly explained in the original manuscript. We appreciate the opportunity to clarify this point. We also recognize that the response to an HFD can vary significantly, influenced by factors such as diets composition, palatability, animal strain, age, protocol duration, and environmental conditions. We have added the following text to the **Discussion** section to address this issue as described bellow:

*The results of this study reveal an interesting and partially divergent finding from the existing literature, as hyperphagia was not observed in animals exposed to the high-fat diet (HFD). Food consumption and caloric inatke were higher in the groups fed a standard diet (SD) compared to those fed an HFD throughout the experimental period (Figs 2A and 2B). However, feeding with an HFD resulted in similar energy efficiency despite lower caloric intake, demonstrating a greater conversion of ingested energy into body mass gain. This phenomenon can be explained by differences in nutrient-induced thermogenesis, favoring fat deposition in HFD-fed animals (10,11). In contrast, SD-fed animals required substantially higher food consumption to achieve comparable energy efficiency. The rapid rise in obesity prevalence can be partially attributed to the widespread availability of highly palatable, energy-dense foods, leading to food intake that exceeds energy requirements. However, previous studies have shown that high-fat diets, when not combined with other components such as sucrose or reinforcing flavors, may be less palatable to rodents, thereby reducing spontaneous intake (1,2).* **(Discussion, pages 18 and 19, last paragraph).**

1. Oliva L, Aranda T, Caviola G, Fernández-Bernal A, Alemany M, Fernández-López JA, et al. In rats fed high-energy diets, taste, rather than fat content, is the key factor increasing food intake: A comparison of a cafeteria and a lipid-supplemented standard diet. PeerJ. 2017;2017(9).
2. Kobi JBBS, Matias AM, Gasparini PVF, Torezani-Sales S, Madureira AR, da Silva DS, et al. High-fat, high-sucrose, and combined high-fat/high-sucrose diets effects in oxidative stress and inflammation in male rats under presence or absence of obesity. Physiol Rep. 2023 Apr 1;11(7).

**It may be good to use a different term for “adiposity index”, since adiposity index is an established term for an index based on hip circumference in humans which may cause confusion.**

Dear Reviewer, thank you very much for your comentary regarding the terminology used. We fully understand that the term "adiposity index" is traditionally employed in human studies, particularly referring to an index based on hip circumference measurements. However, we would like to point out that the use of the term "adiposity index" to describe the ratio of total body fat to body weight in animal models, such as rats, is well established in the scientific literature (1-5)). We very much appreciate the opportunity to explain the use of the term adiposity index in rats. The term adiposity index is widely used in obesity studies to determine body fat in rodents. It is calculated as the sum of the epididymal, retroperitoneal, and perirenal fat depots’ weights divided by the total body weight (g) × 100 and it is expressed as a percentage of adiposity (4-5). Nevertheless, we are not aware of an alternative nomenclature that could replace this term without causing further confusion, but we remain fully open to any suggestions you may have and would be pleased to adjust the terminology according to your recommendations.Thank you again for your valuable feedback, which has greatly contributed to improving the clarity and precision of our manuscript.

1. KOBI, et al. High‐fat, high‐sucrose, and combined high‐fat/high‐sucrose diets effects in oxidative stress and inflammation in male rats under presence or absence of obesity. **Physiological Reports**, v. 11, n. 7, p. e15635, 2023.
2. PIMENTEL CORDEIRO, et al. Resistance to obesity prevents obesity development without increasing spontaneous physical activity and not directly related to greater metabolic and oxidative capacity. **Plos one**, v. 17, n. 8, p. e0271592, 2022.
3. CARDOSO, et al. A high-fat diet induces cardiac damage in obesity-resistant rodents with reduction in metabolic health. **Cell. Physiol. Biochem**, v. 57, p. 264-278, 2023.
4. JEYAKUMAR S.M., LOPAMUDRA P., PADMINI S., BALAKRISHNA N., GIRIDHARAN N., VAJRESWARI A. Fatty acid desaturation index correlates with body mass and adiposity indices of obesity in Wistar NIN obese mutant rat strains WNIN/Ob and WNIN/GR-Ob. **Nutr. Metab.** 2009;6:27. doi: 10.1186/1743-7075-6-27.
5. BASTÍAS-PÉREZ M., SERRA D., HERRERO L. Dietary Options for Rodents in the Study of Obesity**. Nutrients.** 2020 Oct 22;12(11):3234. doi: 10.3390/nu12113234

**Were all the lipid and hormone parameters measured from blood collected during killing? It is stated that rats were 8h fasted for lipid analysis, but no such information is given for the hormone measurements. Also consider that glycemia and, hence, also insulinemia are severely deranged in response to anesthesia.**

Dear Reviewer, we sincerely appreciate your valuable comments and agree that this information was not clearly stated in the text. We would like to clarify that all lipid and hormonal parameters were obtained from blood samples collected after euthanasia, during which the animals were subjected to a fasting period of 6 to 8 hours prior to collection. As requested, the sentence was rewriteen and this information was added in the **Materials and Methods** section to ensure it is clear that, for both lipid and hormonal analyses, the animals underwent this fasting protocol as described below.

*To analyze the lipid and hormonal profiles, the animals were fasted for 6 to 8 hours. Serum concentrations of total cholesterol (TC), high-density lipoprotein (HDL) and low-density lipoprotein (LDL) were determined using specific kits (Bioclin Bioquímica®, Belo Horizonte, Minas Gerais, Brazil and Synermed do Brasil Ltda, São Paulo, Brazil). The results of the colorimetric enzymatic tests were determined using a BS200 automated biochemical analyzer (Mindray do Brasil - Comércio e Distribuição de Equipamentos Médicos Ltda, São Paulo, Brazil). Hormone concentrations of leptin, glucagon and insulin were determined by the ELISA method, using specific kits (Linco Research Inc, St. Louis, MO, USA), according to the manufacturer's instructions. The readings were taken using a microplate reader (Spectra MAX 190, Molecular Devices, Sunnyvale, CA, USA).* **(Material and Methods, Biochemical and hormonal profile, pages 9 and 10, first paraghaph).**

In relation to question about ***“Also consider that glycemia and, hence, also insulinemia are severely deranged in response to anestesia”,*** we would like to clarify that the glucose tolerance test, that is, the assessment of the glycemic profile, was performed without the use of anesthesia. Blood sampling for glycemic analysis occurred prior to euthanasia, and the animals were subjected only to a 6-hour fasting period before these procedures. In contrast, analyses of lipid and hormonal parameters were conducted using blood samples collected after the euthanasia of the animals. As mentioned by Reviewer and several studies from literature, anesthesia can influence metabolic variables such as glycemia and insulinemia (1,2). However, we strictly followed the guidelines established by our national animal experimentation ethics committee, which mandates the use of appropriate anesthesia to minimize animal suffering, even when aware of the potential impacts on metabolic parameters. The use of anesthesia is strongly advised and supported by national regulations (CONCEA – National Council for the Control of Animal Experimentation).

Furthermore, although previous studies recognize the potential interference of anesthesia on metabolic parameters, they still validate the comparison between experimental groups, provided that all animals are subjected to the same anesthetic protocol. In our study, all animals underwent the same standardized anesthesia protocol prior to blood collection, thereby minimizing potential experimental biases and ensuring comparability between groups.

In this context and considering the Reviewer's point into account, we will investigate the effect of these anesthetics in future studies in our laboratory. We thank the Reviewer and inform him that this suggestion will be applied in our studies.

1. JOHANNE A WINDELØV J., PEDERSEN J., HOLST JJ. Use of anesthesia dramatically alters the oral glucose tolerance and insulin secretion in C57Bl/6 mice. **Physiol Rep.** 2016 Jun;4(11):e12824. doi: 10.14814/phy2.12824.
2. Bhathena SJ. Comparison of effects of decapitation and anesthesia on metabolic and hormonal parameters in Sprague-Dawley rats. **Life Sci.** 1992;50(21):1649-55. doi: 10.1016/0024-3205(92)90451-t.

**Figs. 1, 2, and 3 showing the protocol details could be fused into Fig.1A,B,C. This might be clearer for the reader.**

Dear Reviewer, we sincerely appreciate your valuable suggestion and we totally agree that the merger of Figures 1, 2 and 3 into a single figure can clarify information about protocol for the reader. Thus, as requested the Figures 1, 2 and 3 were reformulated to provide a clearer presentation of the protocol. The updated figure and its corresponding legend are provided below.

**Fig 1.** Experimental design. (A) Schematic representative of the group classification criterion by tercile based on animal body weight; (B) Progressive maximal effort test; (C) Experimental protocol.

**The term “Killing” might be more appropriate than “Euthanasia” (which means killing to prevent suffering).**

Dear Reviewer, we sincerely appreciate your valuable comments regarding the terminology used in our manuscript. After careful consideration of the relevant normative and ethical frameworks, we respectfully maintain the use of the term "euthanasia" to describe the procedures employed in our study.

According to the *Euthanasia Guide for Animals Used in Research and Teaching* published by the Animal Ethics Committee of the Federal University of São Paulo (1), humane euthanasia is defined as the practice of inducing death in animals through scientifically validated and ethically acceptable methods. It is indicated both when there is irreversible compromise of animal welfare and at the conclusion of scientific activities approved by an Animal Ethics Committee. Even when the primary objective is the collection of biological material, the focus on ensuring minimal pain, stress, and anxiety fully justifies the use of the term "euthanasia" (1).

Additionally, the Brazilian National Council for the Control of Animal Experimentation (CONCEA) emphasizes that an appropriate euthanasia method must ensure the rapid and irreversible loss of consciousness, without causing unpleasant emotional or physical experiences such as pain, distress, fear, or anxiety (2). All procedures performed in our study adhered strictly to these principles, thus supporting the ethical and scientific appropriateness of using the term "euthanasia." We trust that this explanation clarifies our rationale and demonstrates our commitment to the highest standards of animal welfare and scientific rigor. However, we thank the Reviewer for his comment and inform him that we will investigate the suggested use of the word “**Killing**”.

1. Comissão de Ética no Uso de Animais – CEUA – UNIFESP. *Guia de eutanásia para animais em pesquisa e ensino*. 2nd edition. São Paulo: Federal University of São Paulo, 2024.
2. BRASIL. National Council for the Control of Animal Experimentation – CONCEA. *Guidelines for Euthanasia Practices*. Normative Resolution No. 37, February 15, 2018.

**In line 512 it is speculated that resistance to obesity may be associated with increased lipid oxidation. This does not fit with the lack of a difference in RQ between the OP and OR rats.**

Dear Reviewer, we greatly appreciate your careful reading and the insightful comment regarding the potential inconsistency between the speculation of increased lipid oxidation in obesity-resistant animals and the lack of significant difference in the respiratory quotient between the OP and OR groups. We acknowledge that there was a miscommunication in our original discussion. To address this issue, we have revised the text and added the following information in the **Discussion** section:

*In agreement with our findings, Akieda-Asai et al. (2013) (9) observed that OR animals exhibit a transverse adipocyte sectional area similar to control groups, but significantly smaller than that of OP animals. Additionally, the same authors demonstrated that OR animals show lower expression of fatty acid synthase (FAS) and elevated levels of carnitine palmitoyltransferase-1 (CPT-1), suggesting that the OR phenotype is associated with decreased lipogenesis and enhanced lipid oxidation, respectively. However, our findings partially diverge from these observations, as no significant differences in the respiratory quotient (RQ) were detected between OP and OR groups. Moreover, the evaluation of OR animals should consider not only the lower body mass gain and adiposity but also the maintenance of caloric intake comparable to that of OP animals.* **(Discussion, pages 19 and 20, last paragraph).**

**Fig. 7C: Under the given circumstances, the RQ should be between 0.7 and 1.0, but the depicted values are far above this range. Metabolic cages are sometimes not 100% precise in this regard, but the numbers shown here are very far from the appropriate range.**

Dear Reviewer, we sincerely appreciate your comment regarding the respiratory quotient (RQ) values presented in Figure 7C. After carefully re-evaluating the data, we acknowledge that the obtained RQ values are above the typically expected hysiological range (0.7 to 1.0). We believe the issue may be related to the calibration gas of the system, as we had to replace it at the end of the incremental treadmill tests. Therefore, we have decided to remove this analysis from the current manuscript and instead use the basal VCO_2_, basal VO_2_, and RQ values obtained during the incremental test, in which the animals remained on the metabolic treadmill for 10 minutes. A mean of the values obtained during the 10-minute acclimatization period was calculated. The following text will be added to the **Methodology section:**

**Evaluation of Basal Metabolism and Physical Performance**

*To familiarize the animals with treadmill running, rats were subjected to adaptation sessions on a specific rodent treadmill (BONTHER – Ribeirão Preto, SP, Brazil) for five consecutive days, at a speed of 10 m/min for 10 minutes per day. All animals performed the VO_₂max_ test at least 48 hours after the final familiarization session. The assessment of maximal oxygen consumption (VO_₂max_) was conducted using a progressive treadmill protocol adapted from previous studies (29,30), during weeks 22 and 23 of the experimental protocol. Animals were first acclimated to the treadmill for 10 minutes before the beginning of the test, during which basal VO₂, basal VCO₂, and the respiratory quotient (RQ) were measured. Subsequently, the animals underwent a 5-minute warm-up at a low speed (3 m/min). The test started at a speed of 6 m/min, with increments of 3 m/min every 2 minutes (Fig. 1B).*

*The criteria for determining VO_₂max_ included: stabilization of the oxygen consumption curve despite increasing workload, a respiratory exchange ratio greater than 1.05, or when the animal remained on the electric stimulus grid (1.5 µA) without resuming running within 15 seconds. VO_₂max_ was defined as the highest VO₂ value achieved during the test. Additionally, the maximum speed, total distance covered, and test duration were recorded to determine aerobic capacity and physical performance.* **(Material and Methods, Evaluation of Basal Metabolism and Physical Performance, pages 9 and 10)**

Additionally, the text, the figure reformulated, and figure legend will also be added in the text and **Results** section:

*To investigate the relationship between maximal oxygen consumption (VO₂max), physical performance, and phenotypic differences among the experimental groups, we conducted specific metabolic and functional assessments, as illustrated in Figure 6. Initially, we analyzed basal metabolic parameters, including basal oxygen consumption (VO₂basal), basal carbon dioxide production (VCO₂basal), and basal respiratory quotient (RQ) (Figs. 6A–C). However, no significant differences were observed between groups under basal conditions for VO₂ and VCO₂. (Figs. 6A and B). The results also showed that HFD-OP animals exhibited a lower RQ compared to SD-OP animals, indicating a predominance of fat utilization as an energy substrate (Fig. 6C). Nevertheless, no significant differences in RQ were found among the other groups. Additionally, the HFD-OP group showed higher VO₂ compared to the SD-OP group (Fig. 6D). Moreover, OR animals subjected to the HFD displayed greater final running speed and total distance covered compared to OP animals on the same diet (Figs. 6E and 6F). Finally, the SD-OR group demonstrated a longer relative test time compared to the SD-OP group (Fig. 6G), and similarly, the HFD-OR group also exhibited a longer relative test time compared to the HFD-OP group..* **(Results, pages 15 and 16)**

**Fig 6. Evaluation of basal metabolic parameters and physical performance in experimental groups.** (A) Basal oxygen consumption (VO_2basal_); (B) Basal carbon dioxide production (VCO_2basal_); (C) Basal respiratory quotient (RQ); (D) Maximal oxygen volume (VO_2max_); (E) Total speed of the physical performance test (m/min); (F) Total distance covered in the physical performance test (m); (G) Total duration of the physical performance test (min); Groups: standard diet obesity-resistant (SD-OR, n=11), standard diet obesity-prone (SD-OP, n=12), high-fat diet obesity-resistant (HFD-OR, n=9), and high-fat diet obesity-prone (HFD-OP, n=11). Data are expressed as mean ± standard deviation. p < 0.05 – * Significant difference between the 1^st^ and 2^nd^ test, within the group. Two-way ANOVA, followed by Tukey's *post-hoc* test.

**It appears that graph C and D have been swapped in the legend to Fig.8.**

Dear Reviewer, we appreciate your comentary and attention to detail of the graph C and D on Figure 8 ***(new Figure 6)***. Regarding your observation about the swapping of Figures C and D in the legend of Figure 8 ***(new Figure 6)***, we would like to inform you that we have corrected the error. The legend has been adjusted to correctly reflect the correspondence between the graphs and their respective descriptions. The corrected text for the legend of Figure 6 is as follows:

*Fig 6. Evaluation of basal metabolic parameters and physical performance in experimental groups. This is the Fig 6 legend. (A) Basal oxygen consumption (VO_2basal_); (B) Basal carbon dioxide production (VCO_2basal_); (C) Basal respiratory quotient (RQ); (D) Maximal oxygen volume (VO_2max_); (E) Total speed of the physical performance test (m/min); (F) Total distance covered in the physical performance test (m); (G) Total duration of the physical performance test (min); - Groups: standard diet obesity-resistant (SD-OR, n=11), standard diet obesity-prone (SD-OP, n=12), high-fat diet obesity-resistant (HFD-OR, n=9), and high-fat diet obesity-prone (HFD-OP, n=11). Data are expressed as mean ± standard deviation. p < 0.05 – * Significant difference between the 1^st^ and 2^nd^ test, within the group; Two-way ANOVA, followed by Tukey's post-hoc test.* ***(Figure 6 legend).***

**Figure are very poorly pixelated, almost unreadable.**

Dear Reviewer, we appreciate your comment regarding the quality and resolution of the figures submitted. We would like to clarify that the figures were revised and adjusted according to the submission guidelines provided by the journal. For the revised version of the manuscript, we will upload all figure files through the Preflight Analysis and Conversion Engine (PACE) digital diagnostic tool, as recommended. This process will ensure that all figures are submitted in compliance with the journal’s technical standards, guaranteeing appropriate resolution and clarity. Thank you again for your valuable feedback, which will help improve the overall quality of the final submission.

**Reviewer #2: In the current study, the authors evaluated that the “Improved Physical Performance in Obesity-Resistant Rats Compared to Obesity-Prone Rats: Effects of Different Diets and Metabolic Analysis.” overall manuscript is well written.**

Dear Reviewer, we would like to thank you very much for making your time available to review our manuscript and for the final analysis.
