# Supplementary material for: Improved physical performance in obesity-resistant rats compared to obesity-prone rats: Effects of different diets and metabolic analysis
Source: PLoS One. 2025 Jul 7;20(7):e0327670. doi: 10.1371/journal.pone.0327670 (PMC12233250; doi:10.1371/journal.pone.0327670)
Supplement: Attachment — Submitted filename: Response_to_Reviewers_auresp_2.docx [file pone.0327670.s005.docx]

**Plos One**

| **Subject:** | PONE-D-24-57591R1 |
| --- | --- |
| **By**: | Daniel Sesana da Silva, Matheus Corteletti dos Santos, Lucas Furtado Domingos, Jóctan Pimentel Cordeiro, Kiany Miranda, Maria Gabriela Siqueira Tavares, Késsia Cristina Carvalho dos Santos, Ana Paula Lima-Leopoldo, André Soares Leopoldo |
| **Title**: | Improved Physical Performance in Obesity-Resistant Rats Compared to Obesity-Prone Rats: Effects of Different Diets and Metabolic Analysis |

**Comments to the Author**

**Meaningful communication builds on agreement about the terms we use. Maybe a small number of authors in the past have failed to use the term “obese” appropriately, but this is opposed by thousands of publications, which adhered to the definition found in every serious scientific source, which unmistakably defines the term “obesity” is as a state of excessive body fat (not of increased weight with normal body composition; see WHO, NIH, Mayo, etc.). If you insist to use “obese” in a sense that differs from the generally agreed definition, I would at least claim a straightforward statement pinpointing this deviation. This could, e.g., be a sentence that reads as follows or similar: “Note that our rats referred to as obesity-prone grew bigger than the obesity-resistant without a change in body composition and, hence, did not develop obesity according to the usual definition of an increased relative fat mass.”**

Dear Reviewer, we sincerely thank you for the pertinent comment regarding the definition of the term “obesity” and the need for terminological alignment with established scientific guidelines. Accordingly, we agree with the observation that the term “obesity” should be used based on widely recognized criteria, such as the excessive accumulation of body fat, and not merely based on absolute increases in body weight.
As clearly explained by the Reviewer and in accordance with several institutions of great scientific authority such as the World Health Organization (WHO), the National Institutes of Health (NIH), and the Mayo Clinic, which define **“obesity”** as a disease characterized by abnormal or excessive accumulation of body fat that may impair health, and not simply by an increase in body wight or with normal body composition (1-3). In fact, the World Health Organization states that obesity is an abnormal or excessive accumulation of body fat that presents a risk to health (1).

In our study, the classification of animals as obesity-prone (OP) and obesity-resistant (OR) was based on total body weight (HFD-OP 601 ± 79 vs. HFD-OR: 496 ± 27, p <0.05) as the primary criterion, a common practice in previous experimental models (4-6). However, we recognize as a limitation of the present work the lack of a significant difference between the groups regarding the adiposity index — a metric used in actual studies by our group for more precise phenotypic characterization (7-10).

It should be noted that the absence of obesity was not expected. Nevertheless, we showed a statistically significant difference in the cross-sectional area of visceral adipose tissue between the OP and OR groups subjected to the high-fat diet, suggesting that OP animals indeed showed a greater propensity for fat accumulation in specific compartments, even though the global adiposity index did not differ as expected. Despite this relevant finding, we chose and considered it appropriate to add the sentence suggested by the Reviewer to the text.

Therefore, we will follow the Reviewer’s recommendation and include in the manuscript a clear statement indicating that the animals classified as HFD-OP showed greater body mass and more visceral adipose tissue visualized by CSA, but did not present a significant difference in the adiposity index when compared to the HFD-OR group.

The sentence was rewritten and added in the text.

Interestingly, in the present study, animals classified as OP showed greater weight gain than the OR group, without significant changes in overall body composition and, therefore, did not develop obesity according to the standard definition established by the World Health Organization, which characterizes obesity as an abnormal or excessive accumulation of body fat that poses a health risk (34). In this context, OP animals had higher total body weight and increased visceral adipose tissue cross-sectional area compared to OR animals; however, they did not differ significantly in adiposity index, as expected. Thus, in the context of this study, the terms OP and OR refer to relative differences in body mass gain and visceral fat accumulation, rather than the classic definition of obesity based on an overall increase in body fat. Nevertheless, the differences observed in visceral fat accumulation and body mass, together with other relevant metabolic and functional results, support the scientific relevance of the experimental model and the reported results. *(Discussion, page 17, second paragraph).*

1- WHO. **Obesity and overweight**. Geneva: WHO, 2024. Available at: <https://www.who.int/news-room/fact-sheets/detail/obesity-and-overweight>. Accessed on: June 3, 2025.
2- NIH. **Overweight & Obesity Statistics**. Bethesda: NIH, 2022. Available at: <https://www.niddk.nih.gov/health-information/health-statistics/overweight-obesity>. Accessed on: June 3, 2025.
3- MC. **Obesity**. Rochester: Mayo Clinic, 2024. Available at: <https://www.mayoclinic.org/diseases-conditions/obesity/symptoms-causes/syc-20375742>. Accessed on: June 3, 2025.
4- Buckley TN, Omotola O, Archer LA, Rostron CR, Kamineni EP, Llanora JD, Chalfant JM, Lei F, Slade E, Pendergast JS. High-fat feeding disrupts daily eating behavior rhythms in obesity-prone but not in obesity-resistant male inbred mouse strains. Am J Physiol Regul Integr Comp Physiol. 2021 May 1;320(5):R619-R629. doi: 10.1152/ajpregu.00150.2020.
5- Zhang H, Chen S, Yang L, Zhang S, Qin L, Jiang H. Distinct Gut Microbiota and Arachidonic Acid Metabolism in Obesity-Prone and Obesity-Resistant Mice with a High-Fat Diet. Nutrients. 2024;16(11):1579. doi: 10.3390/nu16111579.
6- Lu Y, Li Y, Sun Y, Ma S, Zhang K, Tang X, Chen A. Differences in energy metabolism and mitochondrial redox status account for the differences in propensity for developing obesity in rats fed on high-fat diet. Food Sci Nutr. 2021;9(3):1603-1613. doi: 10.1002/fsn3.2134.
7- Milhem F, Skates E, Wilson M, Komarnytsky S. Obesity-Resistant Mice on a High-Fat Diet Display a Distinct Phenotype Linked to Enhanced Lipid Metabolism. Nutrients. 2024;16(1):171. doi: 10.3390/nu16010171.
8- Belobrajdic DP, King RA, Christophersen CT, Bird AR. Dietary resistant starch dose-dependently reduces adiposity in obesity-prone and obesity-resistant male rats. Nutr Metab (Lond). 2012;9(1):93. doi: 10.1186/1743-7075-9-93.
9- Cardoso JC, Martins VVP, Madureira AR, Sales ST, Filetti FM, Corrêa CR, Nogueira BV, Lima-Leopoldo AP, Leopoldo AS. A High-Fat Diet Induces Cardiac Damage in Obesity-Resistant Rodents with Reduction in Metabolic Health. Cell Physiol Biochem. 2023;57(4):264-278. doi: 10.33594/000000642.
10- Pimentel Cordeiro J, da Silva DS, Torezani-Sales S, Madureira AR, Claudio ERG, Bocalini DS, Lima-Leopoldo AP, Leopoldo AS. Resistance to obesity prevents obesity development without increasing spontaneous physical activity and not directly related to greater metabolic and oxidative capacity. PLoS One. 2022;17(8):e0271592. doi: 10.1371/journal.pone.0271592.

**And the sentence in lines 185,186 seems incomplete: "The adiposity 185 index was calculated by dividing total body fat by final body mass and multiplying the result by ??? (24,25)."**

Dear Reviewer, thank you for your valuable comment. We appreciate your observation and sincerely apologize for the confusion caused by the incomplete sentence. As requested, the information was added in the text and the sentence was rewritten as described below.

*The nutritional profile was determined by analyzing body weight (BW), body fat (BF) and adiposity index (AI). The BW was measured weekly, and the amount of body fat was determined by the sum of epididymal, retroperitoneal and visceral (mesenteric fat) fat deposits. AI was calculated by the formula = total body fat/ final body weight, multiplied by 100 (24,25).* ***(Materials and Methods, Nutritional assessment, page 7)***

**Reviewer #2: As per the instructions, all suggestions were incorporated in the revised manuscript "Improved Physical Performance in Obesity-Resistant Rats Compared to Obesity-Prone Rats: Effects of Different Diets and Metabolic Analysis"**

Dear Reviewer, we would like to thank you very much for making your time available to review our manuscript and for the final analysis.
